# Expanded Phenotype of the *Cln6^nclf^* Mouse Model

**DOI:** 10.3390/cells14090661

**Published:** 2025-04-30

**Authors:** Victoria Chaoul, Sara Saab, Omar Shmoury, Ramy Alam, Lynn Al Aridi, Nadine J. Makhoul, Jihane Soueid, Rose-Mary Boustany

**Affiliations:** 1 Department of Biochemistry and Molecular Genetics, American University of Beirut Medical Center, Beirut 1107 2020, Lebanon; vc07@aub.edu.lb (V.C.); sarasaab961@gmail.com (S.S.); oas17@mail.aub.edu (O.S.); ra490@aub.edu.lb (R.A.); lra16@mail.aub.edu (L.A.A.); nm36@aub.edu.lb (N.J.M.); 2 Department of Anatomy, Cell Biology and Physiological Sciences, American Unibersity of Beirut, Beirut 1107 2020, Lebanon; js61@aub.edu.lb; 3 Division of Pediatric Neurology, Faculty of Medicine, American University of Beirut, Beirut 1107 2020, Lebanon; 4 Neurogenetics Program and Pediatric Neurology, Departments of Pediatrics, Adolescent Medicine and Biochemistry, American University of Beirut, Beirut 1107 2020, Lebanon

**Keywords:** CLN6 disease, *Cln6^nclf^* mice, apoptosis, neurodegeneration, motor deficits, vision loss, retinal degeneration, astrogliosis

## Abstract

Neuronal ceroid lipofuscinoses (NCLs) are a group of autosomal recessive neurogenetic disorders caused by mutations in 14 different genes. CLN6 disease manifests as variant late-infantile NCL (vLINCL) or as an adult variant. In childhood, symptoms include speech delay, vision loss, cognitive and motor decline, seizures, and early death. An in-depth characterization of a naturally occurring Cln6 mutant mouse (*Cln6^nclf^*) is presented, with implications for translational research. The expanded phenotype provides data showing early death, vision loss, and motor deficits in male and female *Cln6^nclf^* mice. Diminished visual acuity in *Cln6^nclf^* mice was noted at 28 weeks of age, but the pathological loss of retinal layers began as early as 2 weeks or postnatal day 14 (P14). Apoptosis was confirmed by TUNEL staining in the *Cln6^nclf^* mouse brain at P8 and in the retina at P12. A peak in glial fibrillary acidic protein (GFAP) expression was established as a normal developmental phenomenon in the wild-type and *Cln6^nclf^* mouse brain cerebellum and the CA2–CA3 regions of the hippocampus at P8. In *Cln6^nclf^* mice, GFAP levels were elevated at P12 in the cerebellum and hippocampus. In the retina, a developmental peak in gliosis was absent, with increased astrogliosis noted at P6 and P8 in female and male *Cln6^nclf^* mice, respectively. This highlights the lack of a sex-dependent response in wild-type mice. These novel data position the *Cln6^nclf^* mouse model as a useful tool for screening potential therapeutics for human CLN6 disease.

## 1. Introduction

Neuronal Ceroid Lipofuscinoses (NCLs) are a heterogeneous group of rare inherited neurogenetic diseases with an onset spanning from infancy to adulthood. This group comprises 14 different monogenic variants, which are inherited in an autosomal recessive fashion, except for an autosomal dominant form of adult Kufs disease (CLN4 disease) [1]. The incidence of NCLs is estimated at 1 in 100,000 live births, with significant geographic and ethnic variability [2,3]. Clinical manifestations include speech delay, retinopathy with vision loss, developmental regression, progressive cognitive decline, and early death [4,5]. A common hallmark is the accumulation of subunit c of mitochondrial ATP synthase in various tissues, primarily within neuronal lysosomes but also in non-neuronal tissues [6,7].

The CLN6 gene in humans and *Cln6^nclf^* mice is 90% identical. It encodes a highly conserved [8,9] endoplasmic reticulum (ER) membrane protein consisting of 311 amino acids in humans and 308 amino acids in *Cln6^nclf^* mice [10]. The non-glycosylated CLN6 protein consists of seven transmembrane domains [9] joined by three luminal and three cytosolic loops, with a C-terminus located in the ER lumen and a cytosolic N-terminus that mediates CLN6 retention within the endoplasmic reticulum [11,12]. Mutations in CLN6 cause either adult-onset Type A Kufs disease [13] or variant late-onset NCL (vLINCL), with symptom onset between 18 months and 8 years of age. Patients with vLINCL present with speech disturbance, retinitis pigmentosa and visual loss, motor impairment, cognitive decline, and seizures [14,15]. The disease progresses to ataxia and spastic quadriplegia [16], with early death occurring between 12 and 15 years of age [17]. Previous studies implicate the CLN6 protein in the anterograde trafficking of lysosomal enzymes as part of a complex with the CLN8 protein in human embryonic kidney 293T (HEK293T) cells, playing a role in maintaining lysosomal function and cellular homeostasis [11]. Additionally, the CLN6 protein interacts with collapsin response mediator protein 2 (CRMP2), suggesting a role in cytoskeletal control [18]. The dysregulation of these processes has broad implications, positioning CLN6 as a critical player in neurodegenerative mechanisms.

One of 72 disease-causing CLN6 mutations that induce vLINCL in humans [19] is a cytosine insertion in exon 4, resulting in a frameshift mutation altering Arg106 to proline and a premature stop codon following a stretch of 25 novel amino acids [20,21] that appears as a 12 kDa truncated immunoreactive band in CLN6 mutant baby hamster kidney (BHK) cell proteins. This mutation does not impact the dimerization of CLN6 polypeptides or their localization [10,21]. Another homologous cytosine insertion mutation at position 307 in exon 4 is responsible for the generation of a naturally occurring *Cln6^nclf^* mouse model [8,9] that recapitulates the human CLN6 disease phenotype and exhibits progressive retinal degeneration, the loss of motor coordination and balance, and memory and learning deficits [20]. The human CLN6 gene maps to chromosome 15q21 [22], and the *nclf* mutant mouse gene to a homologous region on mouse chromosome 9 [8]. Previous studies have described the *Cln6^nclf^* mouse as manifesting neuronal loss and glial reactivity with deposits of subunit C of mitochondrial ATP synthase observed in several cell types [6], the accumulation of autophagosomes, and the dysregulation of metal homeostasis [23,24]. The work described herein provides evidence for previously unrecognized early pathological changes that may impact emerging treatment strategies.

This study aimed to refine the current description of the CLN6 disease mouse model pathology by determining the age at which retinal layer loss occurs in the *Cln6^nclf^* mouse and the precise onset of retinal degeneration. Accelerated apoptosis in the brain and retina is now documented in the *Cln6^nclf^* mouse model, similar to what has been described in the brain and retina of an ovine model and human CLN6 disease [5,25]. This can aid in developing and accurately evaluating targeted therapies aimed at slowing down or preventing neurodegeneration in human CLN6 disease.

## 2. Materials and Methods

### 2.1. Animal Husbandry

All tests and procedures were conducted in compliance with the American University of Beirut Institutional Animal Care and Use Committee (IACUC) guidelines (IACUC approval number: 18-08-497). Homozygous *Cln6^nclf^* (stock number: 002648) and wild-type (WT) C57BL/6J (stock number: 000664) mice were obtained from the Jackson Laboratory. Male and female mice were maintained under a 12 h light/dark cycle and supplied with access to food and water ad libitum. Temperature and relative humidity were monitored and controlled daily within a range of 18–26 °C and 30–70%, respectively. Behavior and general health were assessed weekly, and the ages of deceased animals were recorded.

The same animals that underwent behavioral assessments (n = 7 per genotype and sex) at the later ages of 26–28 weeks were used to assess the number of apoptotic cells in TUNEL and RNA expression studies using real-time PCR (n = 4 per genotype and sex). Retinal pathology studies included the assessment of cell death and gliosis via TUNEL and GFAP staining, respectively, and were performed at the earlier ages of postnatal days P0–P14 (n = 3–4 per genotype and sex).

### 2.2. Experimental Timeline

**Figure 1:**
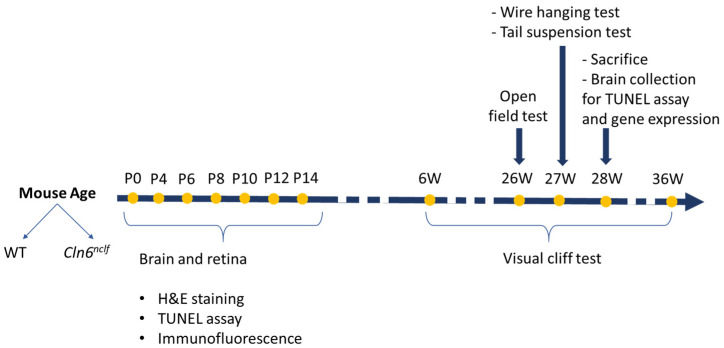
Timeline of experimental procedures performed on wild-type (WT) and *Cln6^nclf^* mice at different ages. Yellow dots indicate time points at which analyses or behavioral tests were conducted. Early postnatal (P) time points (P0, P4, P6, P8, P10, P12, P14) were used for brain and retina analyses, including hematoxylin and eosin (H&E) staining, TUNEL assay, and immunofluorescence. At later ages, behavioral assessments were performed: open-field test at 26 weeks (W), wire hanging and tail suspension tests at 27 W, and visual cliff test between 6 W and 36 W. At 28 W, the mice were sacrificed for brain collection, followed by TUNEL assay and gene expression analysis.

The timeline of experimental work is depicted in Figure 1 for clarity (see Figure 1). 

### 2.3. Genotyping

**Figure 2:**
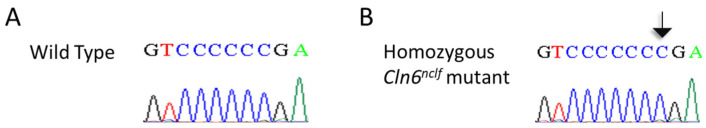
Electropherograms of nucleotide sequences in WT versus *Cln6^nclf^* mutant mouse DNA. (**A**) The wild-type sequence. (**B**) A homozygous 1 bp (C-nucleotide) insertion in exon 4 of *Cln6^nclf^* mouse (c.307insC). The point of variation is indicated by an arrow.

Genomic DNA was extracted from mouse tails. The *Cln6^nclf^* gene was amplified as follows: 30 cycles consisting of polymerase activation (98 °C; 10 s), denaturation (98 °C; 1 s), annealing (60 °C; 5 s), and elongation (72 °C; 15 s). Polymerase chain reaction (PCR) products were run on a 1% agarose gel and visualized using an ultraviolet transilluminator, followed by Sanger sequencing of genomic PCR products (Macrogen-Seoul, Republic of Korea) (Figure 2).

### 2.4. Behavioral Assessment

Behavioral tests were conducted as described in the specific references listed for each of the visual cliff, open-field, tail suspension, and wire hanging tests, without modifications, to assess the performance of male and female WT and *Cln6^nclf^* mice. They were placed in the behavioral room 60 min prior to the onset of tests for habituation. All behavioral tests were performed between 9:00 a.m. and 12:00 p.m. in a brightly lit room. Female and male groups were evaluated separately.

The visual cliff test was employed to assess the visual acuity and depth perception of the animals at different ages, as previously described [26,27]. Separate cohorts of mutant mice were used for testing at various ages. Mice were placed in a Plexiglas box raised 1 m above the ground and positioned halfway over the edge of a table. Its inner surface and ledge consisted of a blue-and-white checkerboard pattern, which emphasized the ledge drop-off and created the illusion of a cliff. Mice were allowed to roam in the chamber freely for a period of 5 min. An overlying camera captured mouse movement and displayed the data in the EthoVisionXT software v.17.5 (Noldus Information Technology, Wageningen, The Netherlands) for further analysis. Time spent over the cliff was recorded. The arena was thoroughly cleaned with 50% ethanol before and after every test session.

Open-field test: To assess mouse locomotor and exploratory abilities, 26-week-old mice were placed in the periphery of a Plexiglas cubic box (50 cm × 50 cm × 30 cm high at the center) and were allowed to explore for 10 min, as previously reported [28]. Walling and rearing events were manually counted. Parameters including total distance traveled, average speed, and time spent in the center/periphery of the arena were recorded by an overlying camera and displayed using EthoVisionXT software v.17.5 (Noldus Information Technology, Wageningen, The Netherlands). The arena was thoroughly cleaned with 50% ethanol before and after every test session.

**Figure 3:**
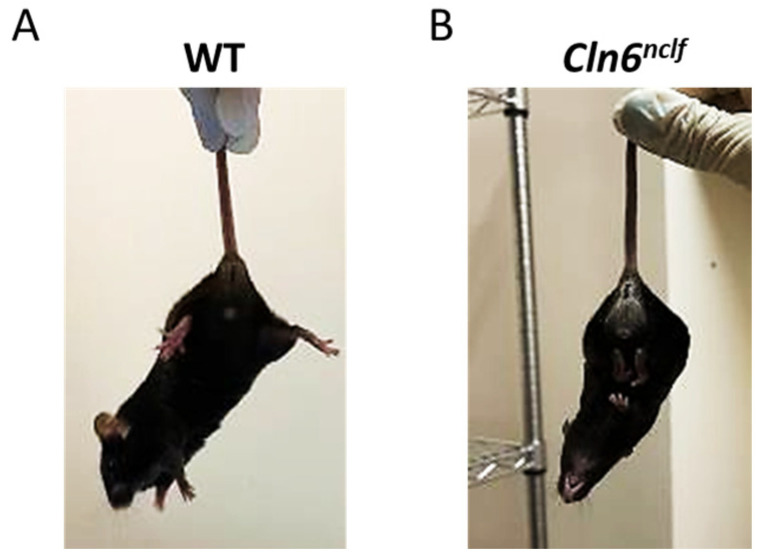
Representative images of the hindlimb clasping reflex in 27-week-old (**A**) WT and (**B**) *Cln6^nclf^* mice. The WT mouse displays a normal response with extended hindlimbs, whereas the *Cln6^nclf^* mouse exhibits a spastic phenotype, characterized by hindlimb retraction and rigidity.

Tail suspension test: To assess mouse spasticity, the hindlimb clasping reflex was used [29,30]. Twenty-seven-week-old mice were grasped by their tails for 30 s and observed for splaying (less spasticity) or flexing (more spasticity) prior to being placed back in their cages, as shown in the included images (Figure 3). Deficits in the ability to splay the hindlimbs and extend the toes were scored based on severity: 3 = both hindlimbs fully clasped; 2 = hindlimbs entirely retracted and touching abdomen for >50% of the observation time; 1 = both hindlimbs partially retracted toward the abdomen for more than 50% of the observation time; and 0 = hindlimbs consistently splayed outward, away from the abdomen, with splayed toes.

Wire hanging test: To determine mouse grip strength and motor coordination [31], the mice were placed in the center of a hanging wire for a period of 90 s. The latency for the mouse to fall off the wire was recorded. This test was repeated five times, allowing 15 min of rest between trials for each mouse.

### 2.5. Histological Processing

Six-month-old mice were deeply anesthetized with a mixture of ketamine and xylazine (100 mg/kg and 10 mg/kg, respectively). Following that, 1 mL of blood was drawn from the inferior vena cava, and the mice were prepared for cardiac perfusion with 30 mL of 4% paraformaldehyde (PFA) in PBS. The mouse brains were removed and then post-fixed in 4% PFA for 2 h at 4 °C and cryoprotected in 20% sucrose. The brains were then embedded in fresh Optimal Cutting Temperature (OCT) compound (obtained from Kaltek S.r.l.) and immersed at −40 °C in isopentane placed in liquid nitrogen for rapid freezing. Coronal brain sections (15 µm) were sliced using a cryostat and mounted on slides prior to staining and stored at −20 °C. For histological studies of the retina, the eyes were removed from WT and mutant mice at postnatal days 0 (P0) through P14, stored in formalin 10% for at least 24 h, and then paraffin-embedded and sectioned. Sagittal sections were cut at 7 µm thickness, mounted on glass slides, and stained with hematoxylin and eosin.

### 2.6. Immunohistochemistry

Paraffin-embedded eyes were deparaffinized by sequentially immersing the slides in xylene (2 × 10 min) followed by graded alcohols (100%, 95%, and 70% ethanol, 2 min each) and finally in distilled water to rehydrate. The sections were washed in PBS (2 × 5 min) and then in PBS-Triton 0.1% (2 × 10 min) to allow permeabilization. Blocking was performed in 10% fetal bovine serum (FBS) diluted in PBS-Triton 0.1% for 1 h, and then the slides were incubated with primary antibodies (anti-rhodopsin (1:500; ab221664) and anti-GFAP (1:500; ab7260) diluted in solution (PBST 0.1–FBS 1%)) overnight at 4 °C. The sections were rinsed in PBS (3 × 5 min), treated with 0.1% Sudan Black for 20 min, and incubated with secondary antibodies for 1 h at RT. The antibodies were washed off, and the sections were counterstained with 1 µg/mL Hoechst (Sigma, St. Louis, MO, USA) and mounted in fluoromount (Sigma, St. Louis, MO, USA) prior to imaging using Leica microscope software v.8.

### 2.7. Terminal Deoxynucleotidyl Transferase dUTP Nick End Labeling (TUNEL) Assay

Apoptotic cells were visualized using the TUNEL Assay Kit BrdU-Red (Abcam; ab66110) according to the supplier’s instructions. Paraffin-embedded brain and eye sections (5-µm) were fixed in a solution of 4% PFA for 15 min, washed with PBS, treated with a 20 µg/mL solution of proteinase K for 5 min at room temperature (RT), washed with PBS, fixed with 4% PFA solution for 5 min at RT, and washed with PBS. The sections were rinsed twice in a wash buffer for 5 min and labeled with the DNA-labeling solution in a dark, humidified 37 °C incubator for 1 h. The sections were washed with PBS to remove the remaining TdT enzyme and incubated with an anti-BrdU-Red antibody for 30 min in the dark at RT. Finally, the slides were covered with Duolink In Situ Mounting Medium with DAPI (Sigma-Aldrich; DUO82040). At least four random microscopic images of each section were obtained using fluorescence microscopy. Three animals were used per experiment, and TUNEL-positive cells were counted using ImageJ software v.1.5.

### 2.8. RNA Extraction from Brain Tissue

Total RNA was isolated from 80 mg of brain tissue using the mirVana™ miRNA isolation kit (Ambion, Austin, TX, USA). The samples were homogenized in a lysis/binding buffer using a motorized rotor–stator homogenizer, and 85 µL of miRNA homogenate was added. The samples were mixed and incubated on ice for 10 min and then centrifuged at 10,000× *g* for 5 min at RT after adding an equal volume of acid–phenol:chloroform. The collected supernatant was mixed with 1.25 volumes of 100% ethanol and centrifuged at 10,000× *g* for 15 s at RT. The samples were washed consecutively with a wash solution and eluted at 95 °C with an elution solution. RNA was quantified using a NanoDrop^®^ ND-1000 spectrometer (Nanodrop Technologies LLC, Wilmington, DE, USA).

### 2.9. Quantitative Real-Time Polymerase Chain Reaction (qRT-PCR)

Complementary DNA was synthesized from 1 µg of RNA via reverse transcription using RevertAid Reverse Transcriptase. qRT-PCR was performed in triplicate with specific primers and SYBR green as the fluorescent detection dye (primer sequences listed in Table 1) according to manufacturer and literature protocols. The results were normalized to *β*-actin expression levels. The 2−∆∆Ct method was used to calculate differences in the relative expression levels of target genes normalized to the housekeeping gene *β*-actin.

### 2.10. Statistical Analysis

Statistical analysis was performed using GraphPad Prism 7 software. For two-group comparisons, data were analyzed with Student’s *t*-test. A normality test was performed to determine whether the data followed a normal distribution. Following this, an F-test was performed to assess whether the two populations had equal standard deviations. For populations not meeting this assumption, Welch’s correction was applied. For comparisons involving more than two groups, a two-way ANOVA was conducted to evaluate interactions between factors, followed by Fisher’s LSD test for post hoc analysis. Data are reported as mean ± standard error of the mean (SEM) derived from three animals and biological replicates from each mouse. Differences with *p*-values < 0.05 were considered statistically significant (* *p* < 0.05, ** *p* < 0.01, *** *p* < 0.001, **** *p* < 0.0001).

## 3. Results

### 3.1. Cln6^nclf^ Mice Have a Shortened Lifespan

**Figure 4:**
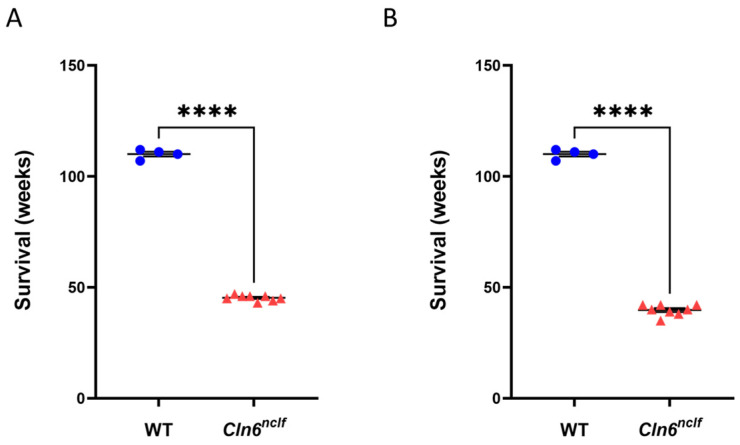
Lifespans of homozygous male and female *Cln6^nclf^* mice. Survival of control (blue circles; n = 4) and homozygous (red triangles; n = 8) (**A**) male and (**B**) female mice. Statistical analysis was performed with Student’s *t*-test. Data are displayed as mean ± SEM. Statistically significant differences in survival between WT and *Cln6^nclf^* mice are indicated as **** *p* < 0.0001.

To determine the impact of the *Cln6^nclf^* mutation on the survival of CLN6 mice, the different groups were monitored for 25 months. Male and female WT mice exhibited normal longevity (110 weeks ± 0.76), while male and female *Cln6^nclf^* mice had a significantly reduced mean survival of 45 weeks ± 0.45 and 39 weeks ± 0.86, respectively (see Figure 4). Also notable is the shorter average lifespan of female *Cln6^nclf^* mice versus male *Cln6^nclf^* mice.

### 3.2. Decreased Cln6^nclf^ Brain Weight

**Figure 5:**
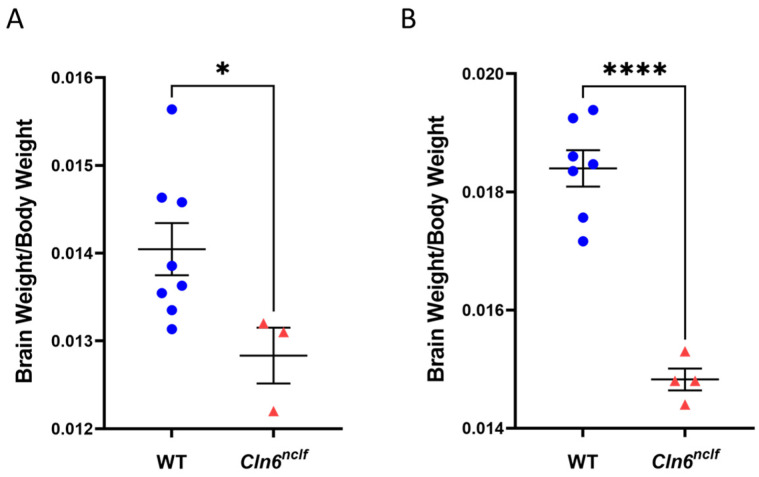
*Cln6^nclf^* mice display reduced brain/body weight ratios. Twenty-eight-week-old (**A**) male and (**B**) female *Cln6^nclf^* mice (n = 3 and n = 4, respectively) exhibit significant decreases in brain/body weight ratios compared to age-matched male and female WT mice (n = 8 and n = 7, respectively). Data are expressed as mean ± SEM. * *p* < 0.05, **** *p* < 0.0001.

The brain and body weights of 28-week-old WT and *Cln6^nclf^* mice were measured prior to animal sacrifice. The findings demonstrate a significant reduction in brain weight/body weight ratios in female and male *Cln6^nclf^* mice compared to age- and gender-matched WT controls (Figure 5A,B below), with a more pronounced decrease in female mice. This decrease in brain mass aligns with observed elevations in apoptotic brain cell numbers, as indicated by the TUNEL assay results.

### 3.3. Retinal Degeneration Precedes Vision Loss in Cln6^nclf^ Mice

**Figure 6:**
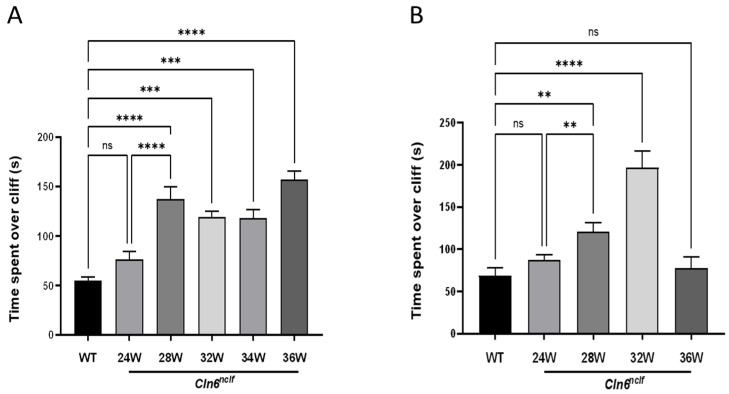
Visual acuity at 28 weeks of age in *Cln6^nclf^* mice. At 6 months of age, *Cln6^nclf^* mice display a significant reduction in visual acuity according to the visual cliff assay. Time spent over the cliff (s) was recorded for WT ages from 6 to 36 weeks (grouped) and (**A**) male and (**B**) female *Cln6^nclf^* mice at developmental stages from 6 to 36 weeks of age. No differences were observed between wild-type and *Cln6^nclf^* mice prior to 24 weeks, and results before this time point are not shown. The data indicate that *Cln6^nclf^* male (n = 10/genotype) and female mice (n = 10 for *Cln6^nclf^* mice; n = 5 for WT) exhibited significant increases in time spent over the cliff compared to their WT counterparts, particularly at 28 weeks, with consistent trends until 34 weeks in males and 32 weeks in females. Please note that ‘ns’ indicates nonsignificant results. Data are expressed as mean ± SEM. ** *p* < 0.01, *** *p* < 0.001, **** *p* < 0.0001.

To determine visual acuity and depth perception in the cohorts of male and female mice, the visual cliff test was performed at time points from 6 to 36 weeks of age. Male *Cln6^nclf^* mice (Figure 6A) displayed a significant increase in time spent over the cliff at 28 weeks (135.51 ± 12.34), indicating a marked decline in visual perception compared to WT mice (55.08 ± 3.9). Female *Cln6^nclf^* mice exhibited a comparable trend, spending significantly more time over the perceived drop-off (120.95 ± 10.71) than age-matched WT mice (68.64 ± 8.98) (Figure 6B). This increase in time was consistent from 28 to 36 weeks in male *Cln6^nclf^* mice and was sustained until 32 weeks in female *Cln6^nclf^* mice. At 36 weeks, however, time spent over the cliff decreased. These findings suggest that male and female *Cln6^nclf^* mice experience critical visual deficits as the disease progresses, although differently.

**Figure 7:**
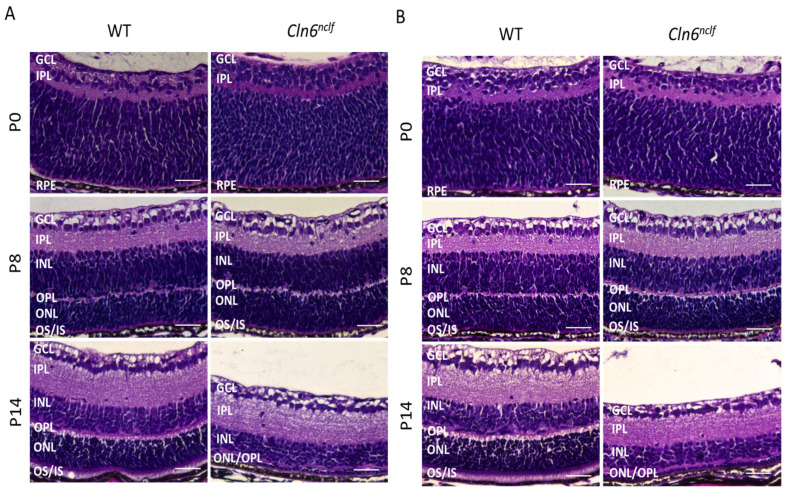
Photoreceptor cell loss and structural changes in the *Cln6^nclf^* mouse retina. Representative histological H&E-stained retinal sections were analyzed to evaluate gross morphological alterations in the retinas of (**A**) male and (**B**) female WT and *Cln6^nclf^* mice. No significant differences in retinal architecture were observed between the WT and *Cln6^nclf^* genotypes at postnatal day 0 (P0) or P8 in male or female mice. At P14, a substantial loss of cells in the ONL and OS/IS layers was evident in male and female *Cln6^nclf^* retinas, confirming an earlier pathological onset of degeneration preceding the behavioral change observed in the visual cliff test at 28 weeks. GCL: ganglion cell layer; IPL: inner plexiform layer; INL: inner nuclear layer; OPL: outer plexiform layer; ONL: outer nuclear layer; OS: outer segment; IS: inner segment; RPE: retinal epithelium layer. Scale bars = 50 µm.

Histological analysis of retinal sections stained with hematoxylin and eosin (H&E) was performed to track the progression of retinal degeneration. The examination of retinal histology in WT and *Cln6^nclf^* mice commenced at postnatal day 0 (P0) and was performed at two-day intervals until postnatal day 14 (P14). From P0 to P12, no significant differences in retinal morphology were detected between WT and *Cln6^nclf^* mice, suggesting that the early retinal architecture remains unaffected in both male and female mice. At P14, significant pathological changes were observed in *Cln6^nclf^* mice, characterized by the substantial loss of cells in the outer nuclear layer (ONL) and the degeneration of the outer (OS) and inner segments (IS) of photoreceptors (Figure 7A,B). This marked pathological deterioration at P14 preceded behavioral deficits identified by the visual cliff test. This emphasizes that retinal cell loss needs to reach a critical threshold before it is behaviorally observed in the visual cliff test, where clinical visual loss is noted at the later age of 28 weeks.

**Figure 8:**
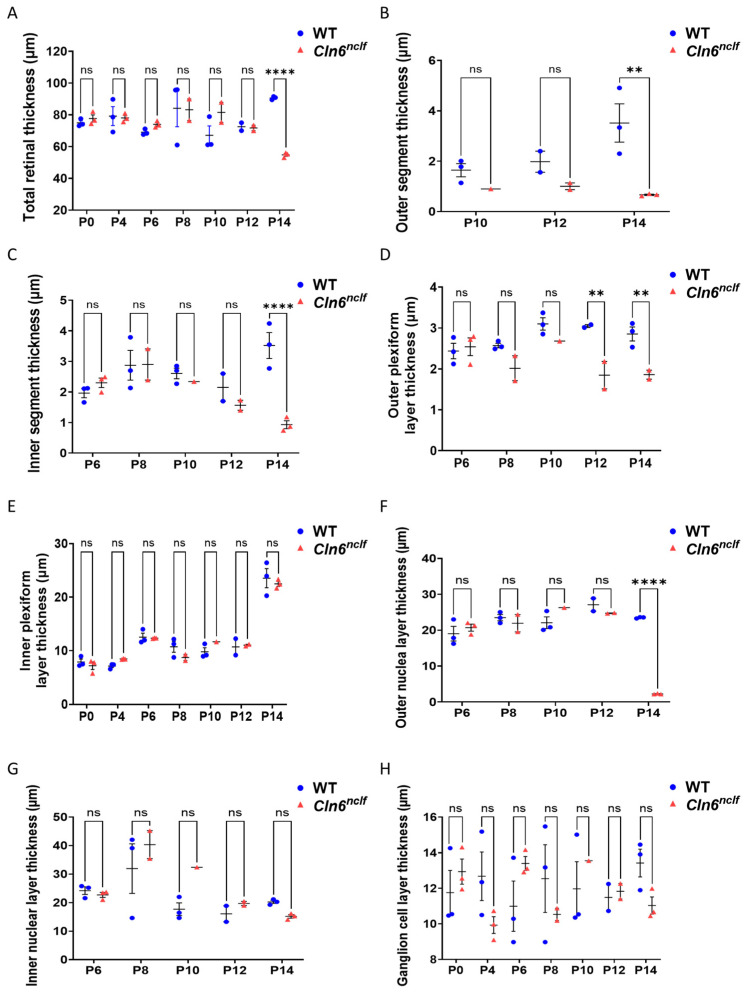

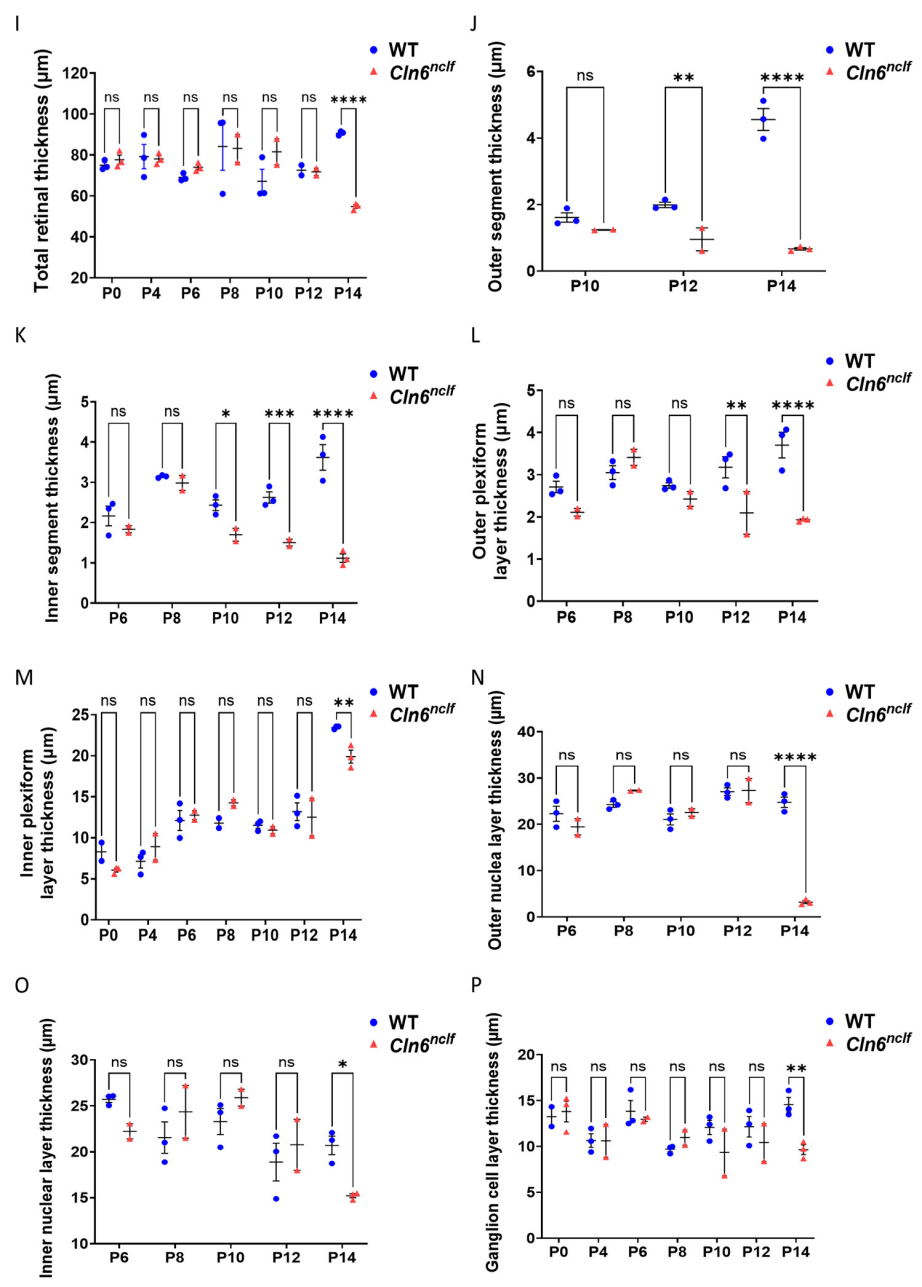
Retinal layer thickness measurements in male and female WT and *Cln6^nclf^* mice. Total retina and individual retinal layer thicknesses (µm) in male and female WT (blue) and *Cln6^nclf^* (red) mice, respectively, are quantitatively depicted in bar graph form at postnatal time points from P0 to P14. (**A**,**I**) Total retinal thickness. (**B**,**J**) Outer segment thickness. (**C**,**K**) Inner segment thickness. (**D**,**L**) Outer plexiform layer thickness. (**E**,**M**) Inner plexiform layer thickness. (**F**,**N**) Outer nuclear layer thickness. (**G**,**O**) Inner nuclear layer thickness. (**H**,**P**) Ganglion cell layer thickness. Data are presented as mean ± SEM. Statistical significance was determined using two-way ANOVA, followed by Fisher’s test. ns = not significant, * *p* < 0.05, ** *p* < 0.01, *** *p* < 0.001, **** *p* < 0.0001.

To further validate the findings from H&E staining, the thicknesses of the total retina and individual retinal layers in male and female WT and *Cln6^nclf^* mice were quantified at multiple developmental time points. Compared to wild-type mice, male *Cln6^nclf^* mice showed significant reductions in the thickness of the total retina (from 90.6 µm to 54.8 µm), outer segment (3.51 µm to 0.66 µm), inner segment (3.52 µm to 0.93 µm), outer nuclear layer (23.5 µm to 2.22 µm), and outer plexiform layer (2.85 µm to 1.86 µm) at P14 (Figure 8A–D,F). No differences were detected from P0 to P12, except for a significant decrease in the outer plexiform layer (from 3.04 µm to 1.8 µm) at P12 in male mice (Figure 8D). In female *Cln6^nclf^* mice, retinal thinning at P14 was more pronounced, affecting the total retina (96.35 µm to 51.69 µm), outer segment (4.55 µm to 0.67 µm), inner segment (3.62 µm to 1.11 µm), outer nuclear layer (from 24.73 µm to 3.19 µm), outer plexiform layer (3.7 µm to 1.93 µm), inner nuclear and plexiform layers (20.7 µm to 15.22 µm and 23.46 µm to 19.9 µm, respectively), and ganglion cell layer (14.56 µm to 9.6 µm) (Figure 8I–P). In females, no significant changes were observed between P0 and P12, except for a decrease in the inner segment starting at P10 (from 2.43 µm to 1.7 µm) and in the outer segment and outer plexiform layer beginning at P12 (1.99 µm to 0.95 µm and 3.17 µm to 2.09 µm, respectively). These results suggest that progressive and sex-dependent retinal degeneration occurs in *Cln6^nclf^* mice, with earlier and more extensive structural impairment in females.

**Figure 9:**
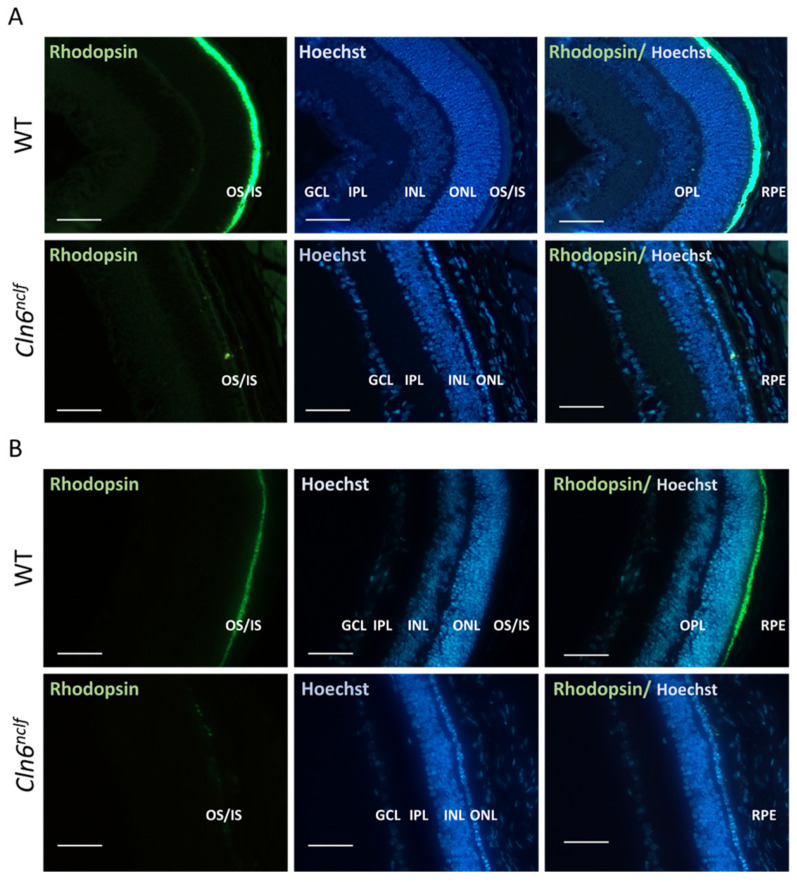
The loss of rod photoreceptor cells in male and female *Cln6^nclf^* mice. Representative micrographs of mouse retina rhodopsin expression (green fluorescence) and a Hoechst counterstain (blue fluorescence) in (**A**) male and (**B**) female WT and *Cln6^nclf^* mice (scale bars = 50 µm). At P14, *Cln6^nclf^* mice exhibited a substantial loss of OS and IS layers where rod photoreceptors reside. GCL: ganglion cell layer; IPL: inner plexiform layer; INL: inner nuclear layer; OPL: outer plexiform layer; ONL: outer nuclear layer; OS: outer segment; IS: inner segment; RPE: retinal epithelium layer.

Rhodopsin is a key light-sensitive protein expressed exclusively in rod photoreceptor cells of the outer and inner segments of the retina (OS/IS). Immunohistochemical staining of rhodopsin in retinal sections confirmed the loss of photoreceptor cells in male and female *Cln6^nclf^* mice at P14. These findings indicate a significant reduction in rhodopsin expression (green fluorescence) in male (A) and female (B) *Cln6^nclf^* mice (Figure 9A,B, respectively) compared to age-matched WT mice. This correlates with a severe loss of rod photoreceptors in male and female *Cln6^nclf^* mice starting at P14.

### 3.4. Retinal Astrogliosis and Apoptosis Timeline

**Figure 10:**
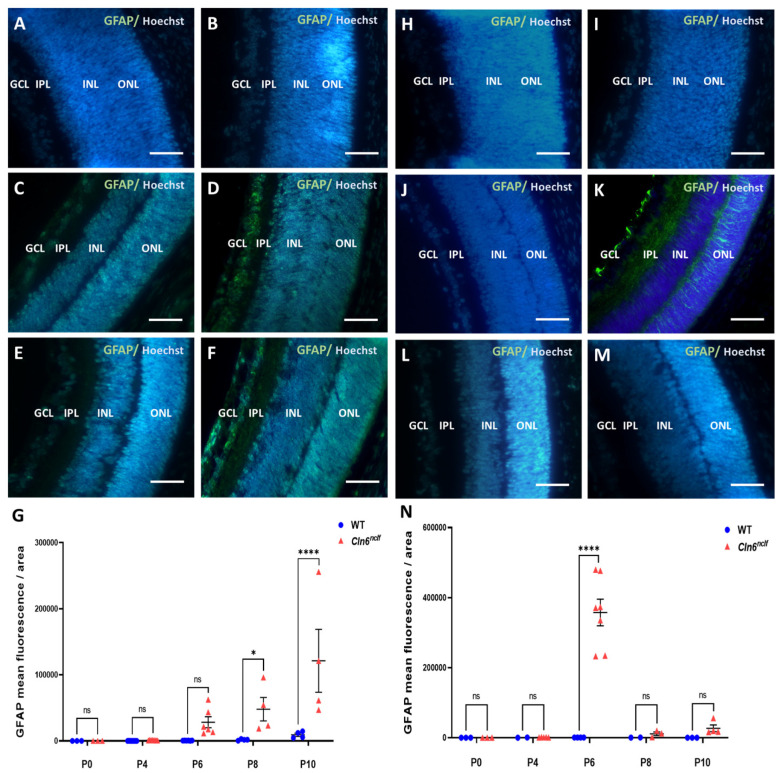
GFAP fluorescence levels in the retinas of *Cln6^nclf^* and WT mice. Representative images of GFAP expression (green fluorescence) in the retinas of male WT and *Cln6^nclf^* mice at postnatal days P0 (**A**,**B**), P8 (**C**,**D**), and P10 (**E**,**F**). Representative images of GFAP expression in the retinas of female WT and *Cln6^nclf^* mice at postnatal days P0 (**H**,**I**), P6 (**J**,**K**), and P10 (**L**,**M**). (**G**) The quantification of GFAP fluorescence intensity demonstrates a significant increase in male *Cln6^nclf^* mice at P8 and P10 compared to WT controls, while female *Cln6^nclf^* mice show a significant increase at P6, followed by a decline at P8 and P10 (**N**). Data are presented as mean ± SEM. Please note that ‘ns’ means nonsignificant. Statistical significance was determined using two-way ANOVA, followed by Fisher’s test (* *p* < 0.05, **** *p* < 0.0001). Scale bars = 50 μm.

Astrogliosis was assessed by measuring the fluorescence levels of glial fibrillary acidic protein (GFAP), a key intermediate filament protein and marker of astrocyte activation. GFAP fluorescence was significantly elevated in male *Cln6^nclf^* mice as early as P8 and remained consistently higher through P10 compared to WT controls (Figure 10G). In contrast, female *Cln6^nclf^* mice exhibited a significant and earlier increase in GFAP fluorescence at P6, followed by a decline in mean fluorescence levels at P8 and P10 (Figure 10N), suggesting distinct temporal patterns of astrocyte activation between the sexes.

**Figure 11:**
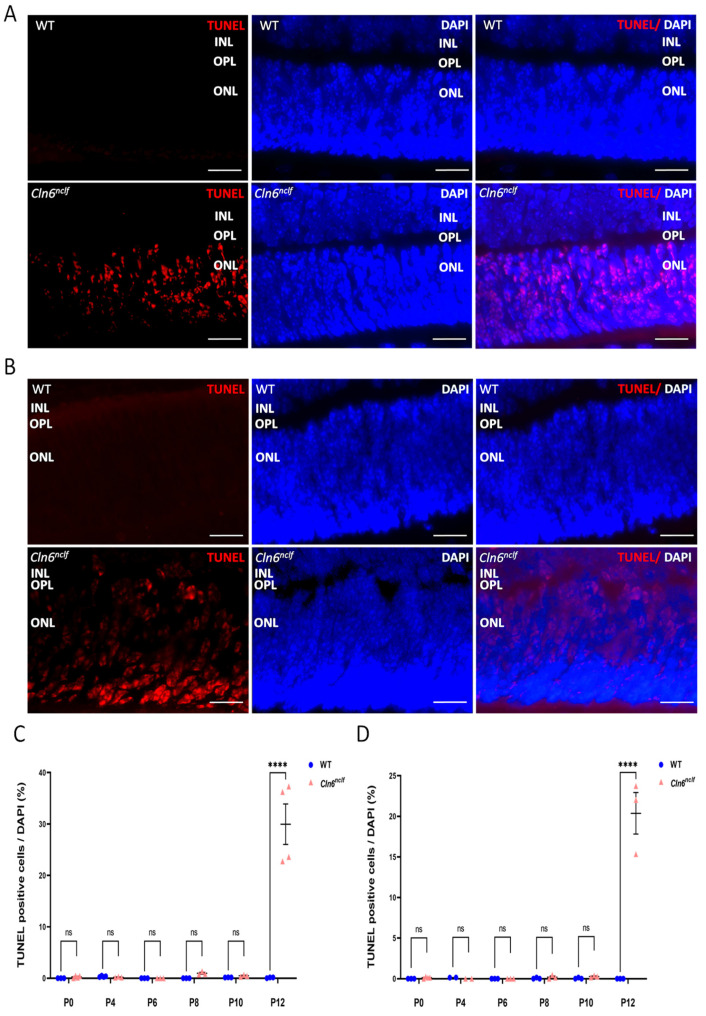
Apoptotic photoreceptor cells in the *Cln6^nclf^* mouse retina. Representative micrographs of retinal sections were evaluated for apoptosis using TUNEL staining (red) and DAPI counterstaining (blue) in (**A**) male and (**B**) female WT and *Cln6^nclf^* mice. Apoptotic cells were restricted to the ONL. At postnatal day 12 (P12), the number of apoptotic cells was higher in *Cln6^nclf^* mice (n = 3; scale bars = 25 µm). TUNEL-positive cells/total cells were counted in (**C**) male and (**D**) female WT versus *Cln6^nclf^* mice at postnatal days P0, P4, P6, P8, P10, and P12. Please note that ‘ns’ means nonsignificant. Data are expressed as ±SEM. **** *p* < 0.0001. ONL: outer nuclear layer; INL: inner nuclear layer; OPL: outer plexiform layer.

To further elucidate the underlying mechanisms of retinal degeneration, a TUNEL assay was performed on retinal sections at various time points spanning from P0 to P12, with intervals of 2 days, to assess the progression of cell death. TUNEL fluorescence, depicted in red, highlights the extensive presence of apoptotic cells at P12 within the outer nuclear layer (ONL) of male (Figure 11A) and female *Cln6^nclf^* mice (Figure 11B) compared to their WT counterparts. Quantitative data confirm that male and female *Cln6^nclf^* mice exhibited a substantially higher number of TUNEL-positive or apoptotic cells, notably at P12, further supporting the hypothesis that increased apoptosis contributes to the loss of photoreceptor cells (Figure 11C,D) observed at P14.

### 3.5. Impaired Exploratory Behavior and Locomotor Activity in Cln6^nclf^ Mice

**Figure 12:**
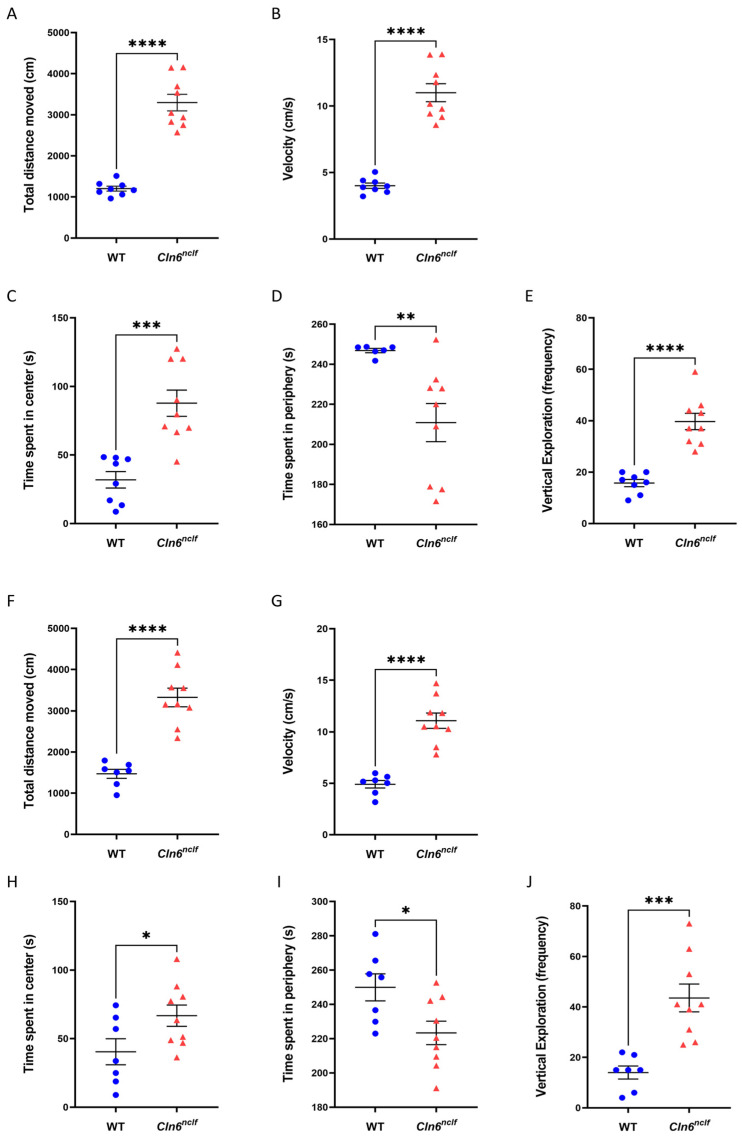
Motor hyperactivity in *Cln6^nclf^* mice, indicated by distance traveled (cm) (**A**,**F**), velocity (cm/s) (**B**,**G**), time spent in the center (s) (**C**,**H**) and periphery (s) (**D**,**I**), and frequency of vertical exploration (**E**,**J**) in 26-week-old male (n = 8 and n = 9, respectively) and female (n = 7 and n = 9, respectively) WT and *Cln6^nclf^* mice. Data are expressed as mean ± SEM. * *p* < 0.05, ** *p* < 0.01, *** *p* < 0.001, **** *p* < 0.0001.

The open-field test was employed to assess exploratory behavior in male and female WT and *Cln6^nclf^* mice. The total distance traveled, average velocity, time spent in peripheral versus central zones of the arena, and vertical exploration, as indicated by rearing and walling behaviors, were documented. Male and female *Cln6^nclf^* mice demonstrated a significant increase in total distance traveled and velocity compared to WT mice (Figure 12A,B,F,G), indicating an increase in overall locomotor activity. Male and female *Cln6^nclf^* mice spent less time in the periphery of the arena (see Figure 12D: 210.86 s and 223.32 s/300 s in males and females, respectively). Additionally, vertical exploration was significantly increased in male and female *Cln6^nclf^* mice (Figure 12E,J).

### 3.6. Spasticity and Motor Dysfunction

**Figure 13:**
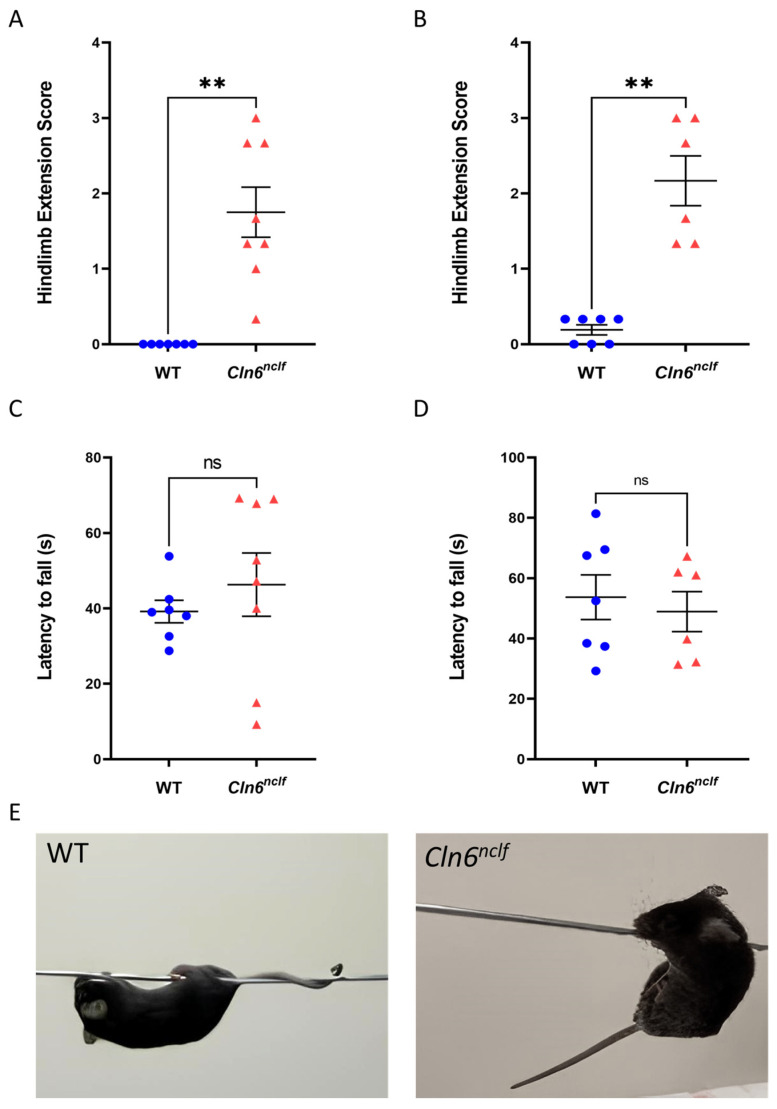
CLN6 deficiency impairs motor control. Twenty-seven-week-old (**A**) male and (**B**) female *Cln6^nclf^* mice (n = 8 and n = 6, respectively) exhibit significantly increased hindlimb clasping compared to WT littermates (n = 7 per sex). No differences were observed in latency to fall (s) off the wire between (**C**) male and (**D**) female *Cln6^nclf^* mice (n = 8 and n = 6, respectively) and their age-matched WT counterparts (n = 7 per sex). Data are expressed as mean ± SEM. ** *p* < 0.01. (**E**) Representative images illustrating the phenotypic differences between WT and *Cln6^nclf^* mice. Please note that ‘ns’ means nonsignificant.

Male *Cln6^nclf^* mice exhibited significant spasticity, with a clasping reflex score 1.75-fold higher than that of WT mice (Figure 13A). Similarly, female *Cln6^nclf^* mice displayed a substantially higher clasping reflex score, up to 2.16-fold, compared to female WT mice (Figure 13B). The wire hanging test was conducted to evaluate grip strength and motor coordination in 27-week-old male and female *Cln6^nclf^* and WT mice (Figure 13C,D). No statistically significant difference in the latency to fall was observed in male and female *Cln6^nclf^* mice versus age- and gender-matched WT mice. *Cln6^nclf^* mice exhibited a clasping phenotype, whereas WT successfully balanced their tail on the wire (Figure 13E).

### 3.7. Astrogliosis in Cln6^nclf^ Mouse Brain

**Figure 14:**
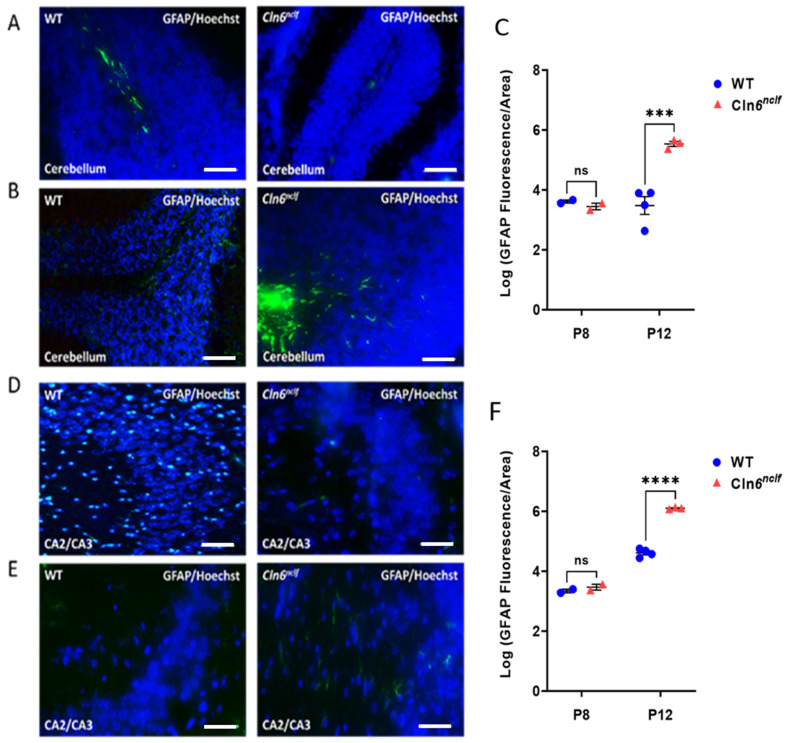
GFAP levels in the cerebella and hippocampi (CA2–CA3) of male *Cln6^nclf^* and WT mice. Representative images of GFAP expression (green fluorescence) in the cerebella of male WT and *Cln6^nclf^* mice at P8 (**A**) and P12 (**B**). (**C**) The quantification of GFAP fluorescence intensity demonstrates a significant increase in the cerebella of male *Cln6^nclf^* mice at P12 compared to WT controls. Representative images of GFAP expression in CA2–CA3 of the hippocampi of male WT and *Cln6^nclf^* mice at P8 (**D**) and P12 (**E**). (**F**) The quantification of GFAP fluorescence intensity demonstrates a significant increase in CA–CA3 of male *Cln6^nclf^* mice at P12 compared to WT controls. Data are presented as mean ± SEM (at P12: 3 *Cln6^nclf^* and 4 WT; at P8: n = 2 per genotype). Please note that ‘ns’ means nonsignificant. Statistical significance was determined using two-way ANOVA, followed by Fisher’s test (*** *p* < 0.001, **** *p* < 0.0001). Scale bars = 50 μm.

GFAP fluorescence staining was used to assess astrogliosis in male *Cln6^nclf^* and wild-type (WT) mice from P0 to P12. No changes were noted from P0 to P6, and results in this timeframe are not shown. At P8, GFAP expression levels were elevated in *Cln6^nclf^* and WT mice, with no significant differences observed in the CA2–CA3 regions of the hippocampus or in the cerebellum between genotypes (Figure 14A–C). A significant increase in GFAP expression was detected at P12, highlighting glial activation in *Cln6^nclf^* mice at this age (Figure 14D–F).

### 3.8. Increased Apoptosis in Cln6^nclf^ Mouse Brain

**Figure 15:**
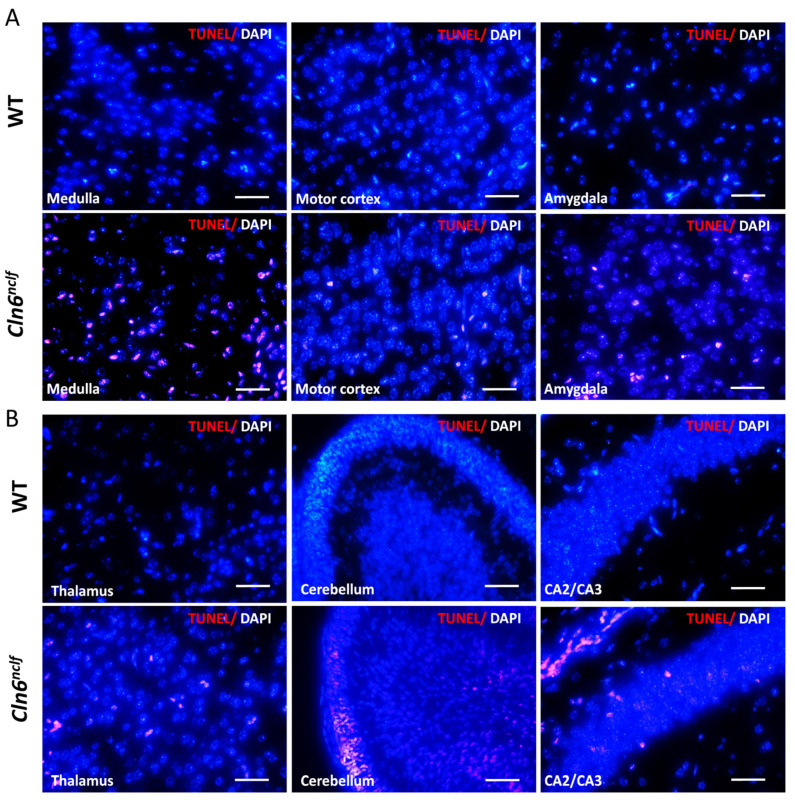

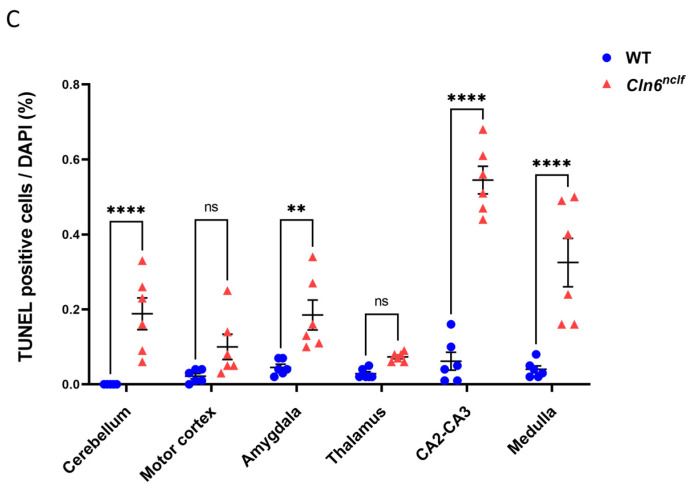
Apoptotic cells in the male *Cln6^nclf^* mouse brain at P8. (**A**,**B**) Representative images of TUNEL assay staining in various brain regions of male mice at postnatal day P8, including the medulla, motor cortex, amygdala, thalamus, cerebellum, and CA2–CA3 of the hippocampus. TUNEL-positive cells are indicated by red fluorescence (scale bars = 50 µm). (**C**) The quantification of TUNEL-positive cells in brain regions: the graph depicts the average number of TUNEL-positive cells/field of view (mean ± SEM). Data were obtained from five WT and six *Cln6^nclf^* mice. Please note that ‘ns’ means nonsignificant. Data were statistically analyzed using two-way ANOVA, followed by Fisher’s test. ** *p* < 0.01, **** *p* < 0.0001.

**Figure 16:**
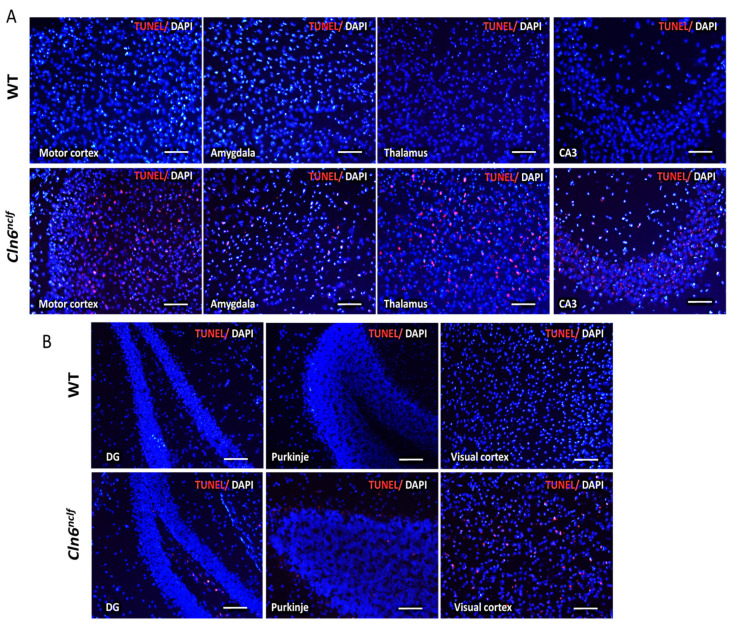

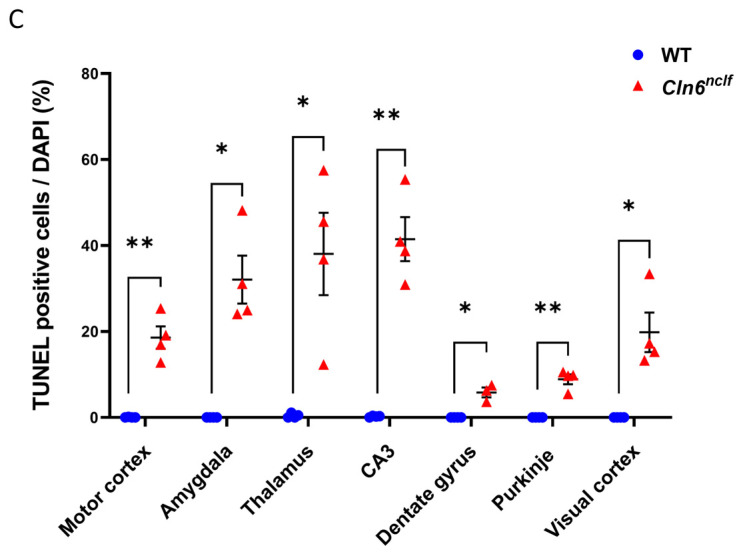
Apoptotic cell quantification in different brain regions of 28-week-old male *Cln6^nclf^* versus WT mice. Representative micrographs of TUNEL staining (red) merged with DAPI counterstaining (blue) in the brains of male WT and *Cln6^nclf^* mice: (**A**) motor cortex, amygdala, thalamus, and CA3; (**B**) the dentate gyrus (DG) of the hippocampus, cerebellar Purkinje cells, and visual cortex (scale bars = 50 µm). (**C**) TUNEL-positive cells were counted in four random fields in brain sections from male WT and *Cln6^nclf^* mice (n = 4/genotype) and are represented as a percentage of TUNEL-positive cells/total cells. Data were statistically analyzed using two-way ANOVA, followed by Fisher’s test. Data are expressed as mean ± SEM. * *p* < 0.05, ** *p* < 0.01.

Apoptosis was evaluated in the brains of WT and *Cln6^nclf^* mice on postnatal days P8, P10, and P12 of life and at 28 weeks (196 days) of age using the TUNEL assay on coronal brain sections. Analysis at P8 confirmed a significant increase in TUNEL-positive cells, indicative of increased apoptosis in the cerebellum, medulla, amygdala, and CA2–CA3 of the hippocampus in male *Cln6^nclf^* mice compared to WT mice (Figure 15A–C). The findings reveal significant increases in apoptotic cell numbers (red fluorescence) within different brain regions of 28-week-old male *Cln6^nclf^* mice (Figure 16A,B), as quantitatively confirmed (Figure 16C) by increases in TUNEL-positive cells in the *Cln6^nclf^* brain compared to the WT brain, specifically in the motor cortex, amygdala, thalamus, CA3 and dentate gyrus of the hippocampus, Purkinje cells, and visual cortex. The thalamus and hippocampus display a high percentage of TUNEL-positive cells/total cells (38.04% and 41.48%, respectively), suggesting that they are more impacted by the disease.

**Figure 17:**
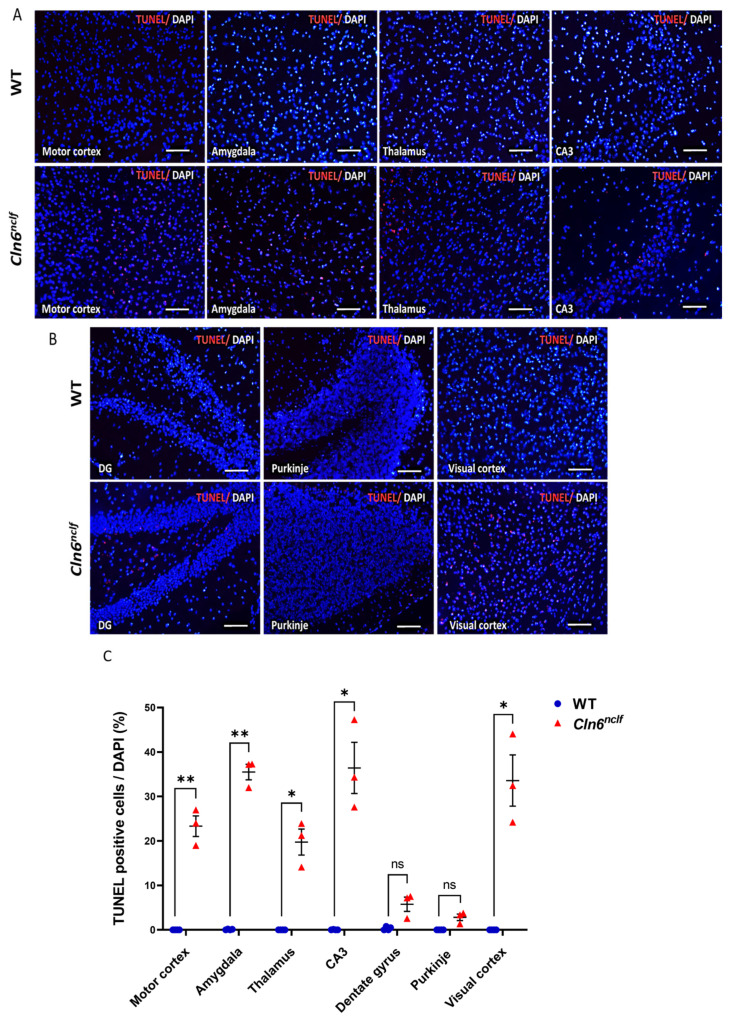
Apoptotic cell death observed in regions of the female *Cln6^nclf^* mouse brain. Representative micrographs of TUNEL-positive cells (red) merged with DAPI-counterstained total cells (blue) in 28-week-old female WT and *Cln6^nclf^* mouse brains: (**A**) motor cortex, amygdala, thalamus, CA3 and (**B**) dentate gyrus (DG) of the hippocampus, cerebellar Purkinje cells, and visual cortex (scale bars = 50 µm). (**C**) TUNEL-positive cells in four random fields of brain sections from WT and *Cln6^nclf^* (n = 4/genotype) mice were counted and are represented as a percentage of TUNEL-positive cells/total cells. Data were statistically analyzed using two-way ANOVA, followed by Fisher’s test. Please note that ‘ns’ means nonsignificant. Data are expressed as mean ± SEM. * *p* < 0.05, ** *p* < 0.01.

The findings confirm that female *Cln6^nclf^* mice exhibit similar pathological features to males. TUNEL-stained brain sections from 28-week-old female *Cln6^nclf^* mice displayed significantly elevated apoptotic cell numbers and intensity compared to age-matched female WT mice in multiple brain regions (Figure 17A,B). This is further confirmed in Figure 17C, with the quantitative analysis confirming more TUNEL-positive cells in female *Cln6^nclf^* mice in the motor cortex, amygdala, thalamus, CA3 in the hippocampus, and visual cortex. No significant differences were identified in the dentate gyrus or cerebellar Purkinje cells in female *Cln6^nclf^* mice. Female mice displayed more apoptotic cells in the amygdala, CA3, and visual cortex (35.49%, 36.42%, and 33.57%, respectively), suggesting increased apoptotic cell death in females.

### 3.9. Comparative Gene RNA Expression Levels in Cln6^nclf^ Versus WT Mice

**Figure 18:**
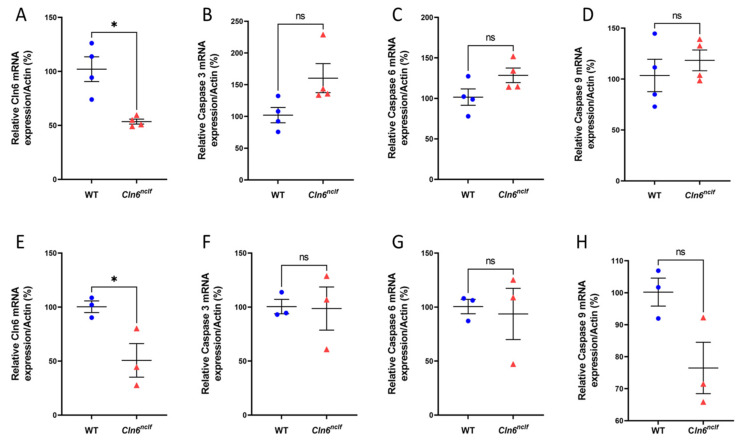
mRNA expression of *Cln6* and caspase mRNAs in WT and *Cln6^nclf^* mouse brains. There is a significant reduction in *Cln6* mRNA expression in the brains of 28-week-old (**A**) male (n = 4) and (**E**) female (n = 3) *Cln6^nclf^* mice compared to age- and gender-matched WT mice (n = 4 and n = 3 respectively). There was no significant difference in gene expression of mRNA caspase levels in male and female *Cln6^nclf^* and WT mice. Specifically, caspase-3 in (**B**) males and (**F**) females, caspase-6 in (**C**) males and (**G**) females, and caspase-9 in (**D**) males and (**H**) females showed comparable expression levels. The levels of mRNA were normalized to actin levels. Please note that ‘ns’ means nonsignificant. Data are expressed as mean ± SEM. * *p* < 0.05.

Pro-apoptotic caspase enzyme mRNA levels in *Cln6^nclf^* mouse brains, determined using qRT-PCR, were 50% lower in 28-week-old male and female *Cln6^nclf^* mice compared to age-matched WT mice (Figure 18A,E). The mRNA levels of caspase-3, caspase-6, and caspase-9 genes were equivalent for male and female *Cln6^nclf^* and WT mice (Figure 18B–D,F–H, respectively).

**Figure 19:**
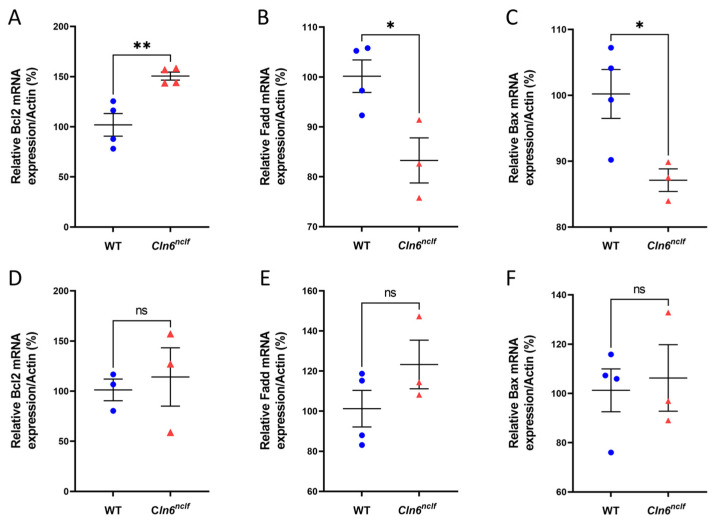
Altered expression of pro- and anti-apoptotic genes in male *Cln6^nclf^* mice. A significant increase was observed in *Bcl-2* expression in the brains of 28-week-old (**A**) male *Cln6^nclf^* (n = 4) versus age-matched WT mice (n = 4). A significant decrease was observed in the expression of *FADD* and *Bax* in the brains of 28-week-old (**B**,**C**) *Cln6^nclf^* (n = 4) compared to age- and gender-matched male WT mice (n = 4). No significant differences were observed in gene expression of Bcl-2, FADD, and BAX between female *Cln6^nclf^* and WT mice (**D**–**F**). Gene levels were normalized to actin values. Please note that ‘ns’ means nonsignificant. Data are expressed as mean ± SEM. * *p* < 0.05, ** *p* < 0.01.

To further examine the impact of the *Cln6^nclf^* mutation on pro- and anti-apoptotic gene expression, anti-apoptotic Bcl-2 and pro-apoptotic BAX and FADD mRNA levels in the brains of 28-week-old male and female *Cln6^nclf^* and WT mice were measured. A significant increase in Bcl-2 mRNA levels was observed in male *Cln6^nclf^* mice compared to WT mice and may explain the decreased apoptosis observed in males (Figure 19A,D). In contrast, concomitant significant decreases in the expression of pro-apoptotic FADD and BAX in male *Cln6^nclf^* but not female mouse brains versus age-matched WT mouse brains were confirmed (Figure 19B,C) and correlated with the shortened lifespan of female versus male affected mice (see Figure 2). The expression levels of the three genes were similar in female *Cln6^nclf^* and WT mice (Figure 19E,F).

## 4. Discussion

The novel data presented provide further insight into the pathogenesis of CLN6 disease in a naturally occurring *Cln6^nclf^* mouse model. The results focus on the timeline and early onset and progression of retinal degeneration, neuronal cell death, and neurobehavioral dysfunction. The implication of apoptosis as a mechanism of neuronal and retinal cell loss in CLN6 disease is confirmed in this mouse model, consistent with observations in the sheep model [5] and in human-CLN6-disease-derived lymphoblasts [25]. The ultimate goal is to inform treatment strategies for patients affected by this condition.

CLN6 disease symptoms in humans are progressive visual loss, motor impairment, cognitive decline, and seizures. Advanced cases manifest ataxia, progressive spasticity, and quadriplegia, culminating in early death. Male and female *Cln6^nclf^* mice died early at 45 and 39 weeks of age, respectively, whereas sex-matched wild-type controls died at 110 weeks. This finding aligns with previous research on other variants of neuronal ceroid lipofuscinoses (NCLs) [32]. Again, progressive neurodegeneration is highlighted as a feature [33]. The disparity between male and female *Cln6^nclf^* mouse longevity and brain weight loss is worth investigating further. Hormonal impacts and dysregulated molecular pathways may skew the immune system toward increased inflammatory responses in females. While estrogen is neuroprotective [34,35], elevated levels may contribute to the accelerated decline observed in females [36,37], perhaps by enhancing autoantibody production [38] and exacerbating the autoimmune response [39]. Additionally, some data show the upregulation of anti-apoptotic Bcl-2 RNA expression and the downregulation of pro-apoptotic FADD and BAX expression in males, consistent with a protective effect, whereas affected female mice have a significantly shorter lifespan, and female patients with CLN6 disease often show increased clinical severity.

An early manifestation of human CLN6 disease is progressive visual loss, leading to blindness. The depth perception abilities of the mice were assessed by observing their explorative behavior toward the cliff in the visual cliff test. Mice with normal retinas exhibit cliff avoidance behavior. *Cln6^nclf^* mice recapitulate the progressive loss of vision in humans, with the near-total loss of visual acuity by 28 weeks, manifesting as significantly more time spent over the cliff. Counterintuitively, female *Cln6^nclf^* mice spent less time over the cliff at 36W. The overall deterioration of older female mice may contribute to this beyond 36 weeks. Previous studies report that *Cln6^nclf^* mice present with photoreceptor cell loss between 13 and 36 weeks of age [20]. Here, a loss of mouse retinal layers as early as postnatal day 14 (P14) or 2 weeks is documented as the significant degeneration of the outer nuclear layer and the substantial loss of rods. Immunostaining for rhodopsin confirmed a marked reduction in expression at 2 weeks or P14 in both sexes, indicating early photoreceptor degeneration. Rothe et al. reported the loss of rod photoreceptor function occurring early at P18, with cone photoreceptor cells being affected later [40], in a time frame consistent with the loss of the outer nuclear layer of the retina. This may mirror what happens in humans with a slow decline in vision, initially manifesting as night blindness and eventually progressing to total vision loss at 28 weeks.

Neuroinflammation, characterized by glial activation and the release of pro-inflammatory cytokines, has been implicated as a critical factor in the development and progression of neurodegenerative disease [41,42,43,44]. Astrogliosis, evidenced by increased GFAP expression, preceded apoptosis in the retina (Figure 10). Specifically, astrogliosis was observed at postnatal day P6 in females and P8 in males, while apoptosis, indicated by TUNEL assays, commenced at P12 in both sexes (Figure 11). In male mice, GFAP levels were elevated at P8 in the ganglion cell layer (GCL), as well as the outer and inner nuclear layers (ONL and INL) of the retina, and remained high through P10, reflecting a sustained gliotic response to retinal degeneration. In contrast, GFAP expression in the female mouse retina peaked at P6 but decreased at P8 and P10. This divergence could be attributed to hormonal influences, as the initial protective role of estrogen contributes to glial activation and a reduction in inflammatory responses in neurodegeneration [45]. Alternatively, the observed reduction in GFAP expression in females may reflect a transition from acute gliosis to a more chronic neurodegenerative process, characterized by diminished glial reactivity with ongoing neuronal loss [46,47]. In the context of neuronal ceroid lipofuscinosis, early glial activation has been observed in other clinical genetic variants [48,49]. In CLN5-deficient mice, retinal degeneration is marked by early-onset photoreceptor death accompanied by retinal inflammation [50]. These findings suggest a pattern where initial glial activation and subsequent inflammation are integral to disease progression. The earlier onset of astrogliosis in female CLN6 mice compared to males indicates potential sex-dependent differences in disease progression [51]. Previous research in CLN8-disease models has reported similar sex-specific variations, with female mice exhibiting an increased disease burden and more accelerated progression [52]. This underscores the importance of the temporal targeting of glial responses in a gender-specific manner in therapeutic strategies aimed at mitigating retinal degeneration.

In this study, a substantial developmental elevation in astrogliosis in the cerebella and hippocampi of wild-type mice and *Cln6^nclf^* mice, with increased GFAP expression in these regions at earlier stages of development, was observed (Figure 14C,G). This finding aligns with previous studies indicating that reactive astrogliosis is a natural part of brain development, particularly in the hippocampus and cerebellum, which undergo significant morphological and functional maturation during postnatal development [53,54]. In fact, studies have demonstrated that astrogliosis occurs as a response to developmental cues, synaptic remodeling, and injury in these regions, and this response typically peaks during specific windows of brain maturation [55,56,57,58,59]. The results presented support the notion that reactive astrogliosis, even in normal conditions, can be region-specific and may be elevated during necessary, key developmental stages and may not be reflective of its contribution to cell death in the brain.

At P12, a significant increase in GFAP levels was observed, specifically in the cerebellum and hippocampus of *Cln6^nclf^* mice. This increase is consistent with the apoptosis noted at P8 at this age in these regions, with higher rates of cell death detected at this time point (Figure 15C). The significant elevation in GFAP expression at P12 suggests that astrogliosis in the CLN6 mouse model may be a response to the earlier apoptotic contribution to neurodegenerative processes associated with the disease, which is corroborated by the increase in apoptotic markers. In *Cln6^nclf^* mice, the astrogliosis response appears to intensify in regions vulnerable to neurodegeneration, such as the hippocampus and cerebellum, which are critical for learning, memory, and motor coordination [60]. Taken together, these results provide evidence of a temporal relationship between apoptosis and astrogliosis in the *Cln6^nclf^* mouse model, reinforcing the idea that astrocytic activation may be a secondary response to the progressive neurodegenerative changes occurring in these regions. Differences in normal and disease biology and progression in the retina and brain of *Cln6^nclf^* mice may necessitate a differential approach in developing timed targeted therapies.

Apoptotic cell death was evident in the retina and multiple brain regions of *Cln6^nclf^* mice (Figure 11, Figure 15, Figure 16 and Figure 17). These findings are consistent with previous studies that demonstrated the presence of apoptotic cells in the retina of an ovine model of CLN6 disease [5]. The TUNEL assay revealed a significant increase in the number of apoptotic cells in various regions of male and female *Cln6^nclf^* mouse brains. Prominent increases in apoptosis in the cerebellum, thalamus, hippocampus, and amygdala (Figure 15C and Figure 16C), occurring as early as P8 and persisting up to 28 weeks, were documented. These results confirm that these regions are preferentially affected, which is reinforced by the significantly diminished brain weight observed in both sexes, indicating that apoptotic cell death is an early contributor to the pathobiology of brain atrophy in *Cln6^nclf^* mice. This widespread apoptosis underscores the aggressive progression of CLN6 disease and its impact on the central nervous system. Similar findings have been reported in other NCL models, with neuronal loss compounded by lysosomal dysfunction, oxidative stress, and mitochondrial impairment [61,62,63].

An array of behavioral tests were conducted to assess strength and coordination. Male and female *Cln6^nclf^* mice exhibit significantly increased spontaneous locomotor activity in response to a novel environment. This hyperactive phenotype was established in a knockout mouse model of CLN3 disease [64] and is confirmed in the *Cln6^nclf^* mouse. This suggests that different NCL subtypes share common pathological and behavioral traits. Hyperactivity in *Cln6^nclf^* mice is identified from their increased propensity to cross the center of the open field with decreased thigmotaxis behavior, best described as contact with a rigid surface directing movement [65], as shown in Figure 12. The increase in locomotor and exploratory activities may suggest that the underlying fear-processing circuitry in the amygdala and other brain regions involved in spatial processing in the hippocampus may be impacted by significant neurodegeneration (Figure 16A,B). The amygdala modulates fear and anxiety [66] necessary for exploratory behavior in mice [65]. Consequently, damage to the amygdala may result in increased exploratory behavior and a reduced propensity for peripheral presence or thigmotaxis. Additionally, damage to the hippocampus can impair spatial learning, leading to an increase in movement and exploration, manifesting as observed hyperactivity. One may intuitively expect *Cln6^nclf^* mice to be hypoactive. The observed hyperactivity is best explained by neurodegeneration in the amygdala and hippocampus depicted in this study (Figure 16 and Figure 17). The tail suspension test in *Cln6^nclf^* mice confirms increased limb spasticity compared to their wild-type counterparts at 27 weeks. Female and male *Cln6^nclf^* mice exhibit comparable falling latency in the wire hanging test. No difference in falling in the wire hanging test was noted; however, an abnormal phenotype of holding onto the wire was observed in *Cln6^nclf^* mice (Figure 13E), perhaps correlating with the confirmed increase in spasticity.

The *Cln6^nclf^* mutation results in a premature stop codon. The significant decrease in *Cln6* mRNA levels is explained by the phenomenon of nonsense-mediated mRNA decay, a cellular mechanism that degrades mRNA molecules containing premature stop codons. This reduces the production of truncated, potentially harmful proteins. Based on results supporting increased apoptosis in neurodegeneration, an increase in the expression levels of caspases is usually expected, indicating the activation of intrinsic/extrinsic apoptotic pathways. However, caspase levels remained unchanged in male and female *Cln6^nclf^* mice compared to wild-type mice. Furthermore, expression analysis revealed the upregulation of Bcl-2 expression and the downregulation of FADD and Bax expression, promoting cell survival, in male mice. The lack of an increase in caspase expression suggests that the classical apoptotic pathway might not be in play. While caspases are crucial for executing apoptosis, their maintained levels do not exclude the activation of other forms of programmed cell death, such as autophagy-dependent cell death, necroptosis, and pyroptosis [67,68,69,70].

Collectively, the findings presented offer further insight into the early pathogenesis of CLN6 disease preceding the appearance of clinical symptoms in *Cln6^nclf^* mice and possibly also in humans, making the case for initiating therapies prior to the appearance of clinical symptoms. The findings indicate that a critical threshold of photoreceptor cell loss must be reached before clinical visual symptoms become evident. While it was possible to establish this threshold for retinal degeneration, linking specific neuronal subpopulation loss to cognitive dysfunction in the brain remains more challenging. This complexity arises from the interplay between different neural circuits in neuronal cell subpopulations that might mask early deficits. Accumulating evidence supports the implication of apoptosis and astrogliosis in the pathophysiology of CLN6 disease, lending support to suggested treatments that regulate these pathways. Understanding the temporal relationship between apoptosis, gliosis, and gender differences in the brain and retina is crucial for developing effective and targeted therapeutic strategies.

Future research should focus on high-throughput genomic and proteomic analyses to pinpoint dysregulated pathways and biomarkers for targeted therapies. Testing pharmacological therapies at this stage could help determine their ability to delay disease progression. These insights deepen our understanding of CLN6 disease and pave the way for advancements in neurodegenerative research and personalized and gender-specific targeted therapeutic development.

## 5. Patents

### 5.1. Application for Method of Treating Batten Disease

Inventor: Rose-Mary Boustany. Provisional filing date: 12 January/1999, Duke Ref. No. 1684. Myers, Bigel Sibley and Sajovek, P.A. (File No. 5405-240 PR). Met filing requirements of US Patent and Trademark Office on 10 July 2002. Assigned Serial No.10/148,859 (U.S. National Phase); Use Patent issued 23 November 2004 US Pat # 6 821 995, expired 23 November 2014.

### 5.2. Functionalized Pyridine Carbamates with Enhanced Neuroprotective Activity

Inventors: P. Trippier, N. Kinarivala, R.-M. Boustany (Texas Tech University and AUB). Application serial number 16/630522. Filing date: 14 July 2017. Receipt date: 13 January 2020, US National Stage under 35 USC 371. International Filing date: 13 July 2018. D-1365 NATL US Official Filing Receipt Ref TTU-1365; File No. TECH: 1166 US. Application Publication 28 January 2021; Publication No. US.-2021-0023064-A1. US Patent issued US 11,369.593 B2

## Figures and Tables

**Table 1:** Mouse primer sequences.

Gene Name	Primer Sequence (5′–3′)	
*Cln6*	Forward	CGGGGACTACTTTCACATGAC
	Reverse	GGGGGACCGCTCAATAAG
*Casp3*	Forward	AGCAGCTTTGTGTGTGTGATTCTAA
	Reverse	AGTTTCGGCTTTCCAGTCAGAC
*Casp6*	Forward	AGACAAGCTGGACAACGTGCC
	Reverse	CCAGGAGCCATTCACAGTTTCT
*Casp9*	Forward	TCCTGGTACATCGAGACCTTG
	Reverse	AAGTCCCTTTCGCACAAACAG
*Bcl-2*	Forward	GTGGATGACTGAGTACCTGAACC
	Reverse	AGCCAGGAGAAATCAAACAGAG
*Bcl-xL*	Forward	AGGTTCCTAAGCTTCGCAATTC
	Reverse	TGTTTAGCGATTCTCTTCCAGG
*Fadd*	Forward	AAGGTGTCTGGTGGGTGTTC
	Reverse	GCATCAGCAAGAGCAGTAGG
*Bad*	Forward	CAGCCACCAACAGTCATC
	Reverse	CTCCTCCATCCCTTCATCC
*Bax*	Forward	CGGCGAATTGGAGATGAACTG
	Reverse	GCAAAGTAGAAGAGGGCAAC
*B-actin*	Forward	ACACTGTGCCCATCTACGAG
	Reverse	ATTTCCCTCTCAGCTGTGGT

## Data Availability

The original contributions presented in this study are included in the article. Further inquiries can be directed to the corresponding author.
